# Revisiting the methionine salvage pathway and its paralogues

**DOI:** 10.1111/1751-7915.13324

**Published:** 2018-10-10

**Authors:** Agnieszka Sekowska, Hiroki Ashida, Antoine Danchin

**Affiliations:** ^1^ Institute of Cardiometabolism and Nutrition Hôpital de la Pitié‐Salpêtrière Paris France; ^2^ Graduate School of Human Development and Environment Kobe University Kobe Japan; ^3^ Institute of Synthetic Biology Shenzhen Institutes of Advanced Studies Shenzhen China

## Abstract

Methionine is essential for life. Its chemistry makes it fragile in the presence of oxygen. Aerobic living organisms have selected a salvage pathway (the MSP) that uses dioxygen to regenerate methionine, associated to a ratchet‐like step that prevents methionine back degradation. Here, we describe the variation on this theme, developed across the tree of life. Oxygen appeared long after life had developed on Earth. The canonical MSP evolved from ancestors that used both predecessors of ribulose bisphosphate carboxylase oxygenase (RuBisCO) and methanethiol in intermediate steps. We document how these likely promiscuous pathways were also used to metabolize the omnipresent by‐products of *S*‐adenosylmethionine radical enzymes as well as the aromatic and isoprene skeleton of quinone electron acceptors.

## Preamble

Appreciating the importance of microbiota is commonplace today. Yet, their analysis still misses truly rational approaches that go beyond correlation studies. To be sure, collecting data at a large scale is so easy that most investigators indulge in a bottom‐up study of big data samples, forgetting that science is hypothesis‐driven. Most studies, while stressing fairly trivial observations such as those identifying as key factors carbon metabolism, variation in microbiota species composition or behaviour related to stressful conditions, rarely take a functional approach. Driving investigation function first would, by contrast, be much more rewarding. Following the synthetic biology stance, where thinking as an engineer is the rule, would allow investigators to look first for key functions. The very richness of microbiomes (microbiota's genes and genomes), with a considerable number of sequences of unknown function – and with another considerable proportion of paralogues of known genes – should provide a rich mine of functional hypotheses. Subsequently, microbiome big data could be used to identify causal relationships between specific microbial functions and the well‐being (or the weak points) of their host. A direct access to such functions is to identify metabolic signatures where specific genes and their cognate protein products, often enzymes, can be understood as contributing to the structuration of the microbiota and its interaction with their host. Besides the heavily trodden carbon metabolism, the fate of the other essential atoms of life, in particular nitrogen, phosphorus and sulphur, must be explored in priority. For the human host, methionine, for example, is an essential amino acid. It is involved in a number of key processes both critical for genetic heredity and for epigenetic heredity. Its metabolic fate, both in the host and in its microbiota must therefore be clarified. Here, we review the way this amino acid is recycled, emphasizing the methionine salvage pathways, variants of which keep being discovered. We hope that this work will help investigators go and identify novel activities not only of metabolites that are linked to the pathway, but for novel enzymes and possibly unknown processes that will allow us to link specific functions to unknown microbial gene sequences.

## A patchwork of historical landmarks in the methionine salvage pathway discovery

The core biosynthetic machinery has been deciphered in‐depth in all three domains of life. By contrast, there are still a number of important metabolic pathways that remain pretty much ignored, despite the fact that their genes span the whole phylogenetic tree. Here, we detail the omnipresent methionine salvage pathway (MSP), first discovered in plants but a key to understand many microbial niches and to develop novel metabolic engineering processes. In contrast to animals, plants do not move. Yet, they often communicate with one another quite efficiently. The way they communicate was long a matter of speculation, until it appeared that they often used gaseous signals (pheromones) to this aim. A significant research effort is now developed to recruit these signals for biotechnological purposes (Pickett and Khan, 2016). Among the variety of those discovered over the years, ethylene plays a major role (Dubois *et al*., 2018). This hydrocarbon gas is both a hormone (allowing intercellular communication) and a pheromone (allowing communication with distant organisms). Gaseous at life's temperature, it is involved in monitoring and triggering growth transitions such as fruit ripening, seed germination, wound healing and shedding of aged leaves, and this can work at a distance. Its presence, first established in 1934 by Richard Gane from an emanation of ripening fruits (for a discussion see Bakshi *et al*., 2015), was subsequently shown to be of physiological significance by a variety of authors (Schuman and Baldwin, 2018 and see Pratt *et al*., 1948 for early experiments). As expected, this observation of considerable biotechnological importance triggered research to uncover ethylene biosynthesis pathways. Working first with apple fruit tissues, Shang Fa Yang at the University of California at Davis established in 1966 that the molecule was derived from methionine (Yang *et al*., 1966). Both methionine and its deaminated form (alpha‐keto‐gamma‐methylthiobutyric acid, KMBA, also abbreviated KMTB) served as precursors. Moreover, these studies established that the endogenous cellular content of methionine was so low that it had to turn over actively for ethylene biosynthesis to reach a significant level.

**Figure 1:**
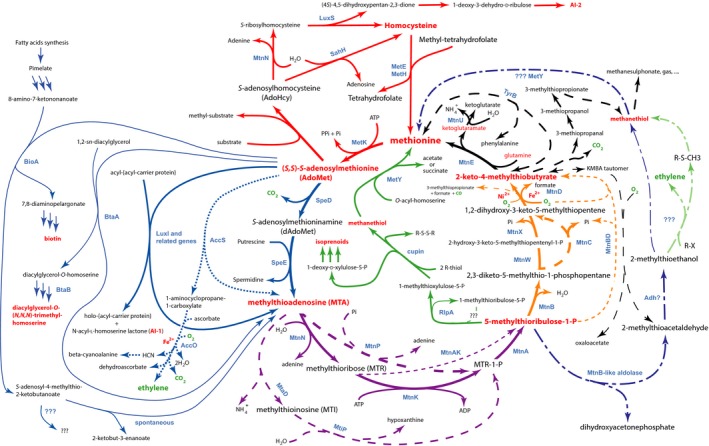
The methionine salvage pathways (see text). Five steps are involved. Red: Methionine is rescued from homocysteine and one‐carbon donors after *S*‐adenosyl methionine has been used in methylation process (not discussed in details here). Methylthioadenosine results from a variety of pathways (blue). It is subsequently metabolized into 5‐methylthioribulose1‐P (purple). This molecule can be processed into several products; in the standard MSP (orange), it is making 2‐keto‐4‐methylthiobutyrate. In other pathways, it produces methanethiol and a precursor of isoprenoids (green) or 2‐methylthioethanol (dark blue). This metabolite results in ethylene production under anaerobic conditions (light green). Finally, (black) methionine is restored after transamination with glutamine. Dashed black arrows also describe how KMBA can be degraded in various organisms.

The corresponding pathway (Baur *et al*., 1971), sometimes named the Yang cycle, was then progressively deciphered with *S*‐adenosylmethionine (AdoMet) at its core. Complete knowledge of the full cycle was achieved in 1987 with the discovery that the conversion of the precursor methylthioribose (MTR) to methionine required l‐glutamine as the most efficient alpha‐amino group donor for the final transamination step (Miyazaki and Yang, 1987). Perhaps surprisingly, oxygen was essential to this conversion, indicating that at least one of the steps in recycling methionine after ethylene production was oxygen‐dependent. Briefly, ethylene synthesis proceeded from methionine via AdoMet, producing 1‐aminocyclopropane‐1‐carboxylic acid (ACC, Fig. 1, dotted light blue arrows), and 5‐methylthioadenosine (MTA), and regenerating methionine at the end of the process in an oxygen‐dependent process.

This MSP was not restricted to plants. Very early on, MTA had been shown to be able to complement methionine auxotrophy in bacteria (Schwartz and Shapiro, 1954). An authentic MSP was progressively deciphered in animals, bacteria and fungi (Backlund and Smith, 1981; Toohey, 1983), and a similar pathway was probably functional in protozoa (Riscoe *et al*., 1989). In *Klebsiella pneumoniae*, 5‐methylthioribose‐1‐phosphate (MTR‐1‐P) was an intermediate as in animals (Tower *et al*., 1991). Subsequently, the *Bacillus subtilis* genome sequence revealed the existence of a MSP in this organism, with the presence of nucleosidase MtnN (YrrU) producing MTR and adenine as by‐products of MTA catabolism, as in the plant pathway (Sekowska and Danchin, 1999). The origin of MTA in these bacteria was established as essentially resulting from spermidine synthesis after AdoMet decarboxylation (Fig. 1, blue arrows; Sekowska *et al*., 2000b). This prompted exploration of the genome sequence with focus on sulphur metabolism (Sekowska *et al*., 2000a). Identification of sulphur islands in bacterial genomes was pointed out as a feature helpful for functional genome annotation and biotechnological applications, suggesting that placing genes randomly in genomes would often have a negative impact on their expression (Rocha *et al*., 2000). In *B. subtilis*, MTR was converted into its phosphorylated derivative MTR‐1‐P by kinase MtnK, an enzyme related to choline kinase (Sekowska *et al*., 2001a). This was concomitant with an unexpected deeper link with arginine metabolism (Sekowska *et al*., 2001b), which also revealed that in microbial eukaryotes such as *Saccharomyces cerevisiae*, a phosphorylase previously identified in human cells or in Archaea (Cacciapuoti *et al*., 1999) could directly produce MTR‐1‐P from MTA, bypassing the nucleosidase/kinase steps, as well as regulate ornithine metabolism (Subhi *et al*., 2003).

Beginning in 1987, the *B. subtilis* sequencing project evolved into a collaboration between European laboratories and Japanese scientists, mainly at the Nara Institute of Science and Technology (NAIST, Ogasawara, 1993). When a gene inactivation experiment monitoring resistance to trifluoromethylthioribose (previously used by Riscoe *et al*., 1989) revealed that MtnW (YrkW) – an enzyme obviously involved in the MSP – was an analogue of ribulose bisphosphate carboxylase/oxygenase (RuBisCO, the most abundant enzyme on Earth), the laboratory of Akiho Yokota at NAIST explored the chemical steps that could allow methionine recycling in *B. subtilis*. Indeed, these investigators had long probed the biochemical steps of photosynthesis, in particular photorespiration, with emphasis on RuBisCO (Yokota and Canvin, 1985). Yokota and colleagues had established the 3D structure of RuBisCO from *Chlamydomonas reihardtii* (Mizohata *et al*., 2002), allowing them to have deep insight into the catalytic activity of the enzyme. In parallel, at Ohio State University, the Robert Tabita laboratory showed that *Rhodobacter capsulatus* possessed two RuBisCO forms, a red‐like and a green‐like form (Horken and Tabita, 1999). In a breakthrough experiment, studying the biochemical properties of the RuBisCO‐like protein (RLP) from *B. subtilis*, Yokota and co‐workers showed that it catalysed the tautomerization (‘enolization’) of 2,3‐diketo‐5‐methylthiopentyl‐1‐phosphate (DK‐MTP‐1‐P) in the MSP (Fig. 1). They also found that its inactivation was rescued by the gene for RuBisCO from the photosynthetic bacterium *Rhodospirillum rubrum* (Ashida *et al*., 2003). Later on, it was shown, however, that this functional complementation was not due to substitution for the *B. subtilis* enzyme activity but possibly by input into another type of salvage pathway, as we will see below (Warlick *et al*., 2012a). These authors further identified all the chemical steps found in *B. subtilis*, and this was subsequently extended to other bacteria, raising unexpected challenges in terms of phylogeny (Sekowska *et al*., 2004). This latter study, while identifying variations on the plant pathway, also showed that in some organisms, the pathway was likely to be different, requiring further studies.

Tabita *et al*. (2007) had previously interpreted the two RLP classes as corresponding to isozymes, until a new phylogenetic classification showed them that there were at least four forms, distributed in the three domains of life, with form IV involved in sulphur metabolism rather than carbon fixation. In complementary studies, John Gerlt at Albert Einstein College of Medicine and his co‐workers explored the mechanistic diversity of the enzymes of the RuBisCO superfamily in the MSP in *Geobacillus kaustophilus* (Imker *et al*., 2007). They further showed that in *R. rubrum*, the enzyme converts 5‐methylthio‐d‐ribulose‐1‐phosphate (MTRu‐1‐P) into a 3:1 mixture of 1‐methylthioxylulose‐5‐phosphate and 1‐methylthioribulose‐5‐phosphate (Imker *et al*., 2008). From this point onward, many studies explored the biochemistry of the enzymes of the MSP, while also uncovering many variants of the pathway and its evolution (North *et al*., 2016). Here, we summarize the present status of this complex metabolic cycle resulting in methionine salvage, following pathways that have been repeatedly rediscovered and combined together via convergent evolution, with many activities still of unknown nature. A main feature of the pathways is that they are organized in most aerobic organisms in such a way as to prevent backward cycling (Fig. 1, black arrows and see below), avoiding methionine degradation while allowing cells to couple sulphur and nitrogen metabolism (Belda *et al*., 2013).

## Biochemical rationale for the methionine salvage pathways (MSP)

**Figure 2:**
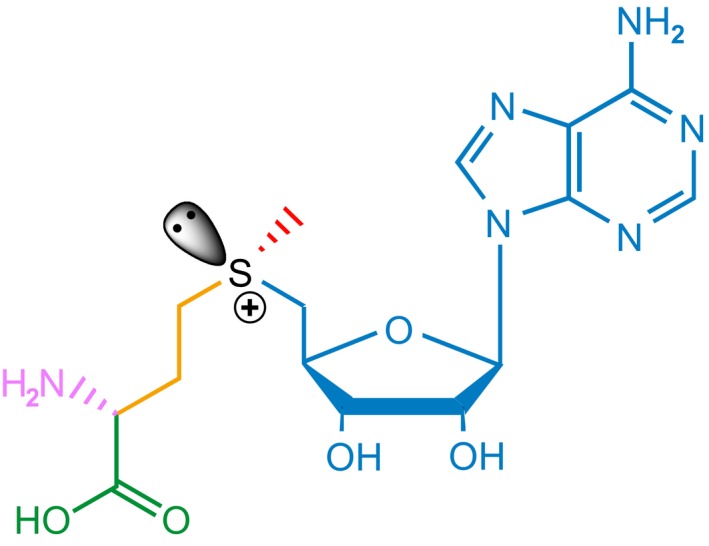
The *S*‐adenosylmethionine molecule. The sulphonium ion allows three types of group transfers: methyl (red), carboxy (green) aminopropyl (orange) and adenosyl (ribosyl) (blue). The purple amino group is used for biotin synthesis in a variety of bacteria.

*S*‐adenosyl methionine is, metabolically speaking, a very costly essential compound. Its synthesis not only draws heavily on the pool of methionine, but it also uses ATP in an unusual way, consuming the energy of three phosphate bonds, while producing both phosphate and pyrophosphate, which is subsequently hydrolysed (Cantoni, 1953; Chen *et al*., 2016). This metabolite has a remarkable structure, with its sulphur atom positively charged (sulphonium). In addition to the chiral carbon at the alpha‐amino position of methionine, the sulphur at the sulphonium centre is also chiral: AdoMet exists in two diastereomers with respect to its sulphonium ion, (*S*,*S*)‐AdoMet and (*R*,*S*)‐AdoMet, where *S* and *R* refer to the conformation at the sulphur and the alpha carbon, respectively. (*S*,*S*)‐AdoMet is the only form synthesized enzymatically. It is also essentially the form used in group transfers. The sulphonium centre is used for three chemical group transfer processes (for a summary see Fontecave *et al*., 2004): methyl (Fig. 2, red), 3‐amino‐3‐carboxypropyl, usually in its decarboxylated form (aminopropyl, Fig. 2, orange and purple), and adenosyl (ribosyl) transfer (Fig. 2, blue). Finally, a wealth of radical AdoMet enzymes produces via reductive cleavage of its C‐S bond a 5′‐deoxyadenosyl‐5′‐radical (AdoCH_2_^·^) that, subsequent to reacting in a variety of chemical processes, usually generates 5′‐deoxyadenosine, which must be scavenged. The corresponding pathways have much in common with MSP reactions. They are discussed at the end of this article.

By far, the most frequent of these steps is methyl group transfer (methylation), resulting in the production of S‐adenosylhomocysteine (AdoHcy), that must be recycled to AdoMet, with the right stereochemistry. By contrast, chemical methylation of AdoHcy yields a racemic mixture of the two AdoMet isomers. Also, under standard physiological conditions, the sulphonium ion of (*S*,*S*)‐AdoMet will racemize spontaneously to the *R* form, and this will tend to waste an essential and costly compound. This introduces an important selection pressure for discovery of a remedy. A homocysteine *S*‐methyltransferase that can use (*R,S*)‐AdoMet, SamT(YitJ), has been identified in *B. subtilis* (Borriss *et al*., 2018) as a counterpart of yeast homologues. It uses both the (*S*,*S*) and (*R*,*S*) forms of AdoMet, with a higher affinity for the (*R*,S) diastereomer (Vinci and Clarke, 2010). This enzyme, and possibly others, will allow the cell to cope with this inevitable accident of metabolism that should be taken into account in biotechnological processes (Danchin, 2017).

Exploration of the methylation pathways in parallel with homocysteine–methionine recycling (reverse transsulphuration and other sulphur scavenging processes; Cho *et al*., 2015) is likely to provide metabolic engineers with relevant tracks. A critical need is that recycling homocysteine to methionine requires methyl donors. The corresponding compounds are quite diverse and vary in different organisms: methyl‐tetrahydrofolate, methyl‐cobalamine and a variety of methylated small molecules [e.g. choline, glycine betaine (Zou *et al*., 2016), *S*‐methylmethionine (Li *et al*., 2016)]. This suggests that disparate related functional associations are likely to operate in microbes living in different niches. In line with this view, we may notice that *B. subtilis* SamT is a bifunctional enzyme associating a methylenetetrahydrofolate reductase [NAD(P)H‐dependent] to (*R,S*)‐AdoMet homocysteine *S*‐methyltransferase. This recycling process (Fig. 1, red arrows) is coupled to a variety of reduced sulphur scavenging processes (Su *et al*., 2016). They have been the subject of many reviews and are not further discussed here (see Seiflein and Lawrence, 2001; Rodionov *et al*., 2004; Hullo *et al*., 2007 for complements).

The second group‐transfer pathway [amino(carboxy)propyl transfer] consumes also a vast amount of AdoMet as, after AdoMet decarboxylation, it is involved in the synthesis of polyamines, metabolites that are fairly abundant in the cell (Cohen, 1998). Direct transfer of the 3‐amino‐3‐carboxypropyl group happens sometimes (Kulikovsky *et al*., 2014). It is used for the modification of anticodon base U34 in *Escherichia coli* phenylalanine tRNA (Nishimura *et al*., 1974) but this does not seem to be widespread. A covalent modification of this tRNA residue is also sometimes resulting from carboxymethyl transfer from an analogue of AdoMet, *S*‐adenosyl‐*S*‐carboxymethyl‐l‐homocysteine (Byrne *et al*., 2013), the fate of which is unknown and not discussed here. These chemical possibilities are, however, important to remember because they underscore the fact that exploration of the huge microbiota datasets presently available remains open to discoveries. For example, 3‐amino‐3‐carboxypropyl group transfer is widespread in eukaryotes as a modification of 18S ribosomal rRNA by ribosome biogenesis factor Tsr3 (Meyer *et al*., 2016). Homologues of Tsr3 (DUF367 family proteins) are widespread in archaea and present in some bacteria as well (Burroughs and Aravind, 2014). In the same way, synthesis of the modified histidine residue diphthamide in archaeal and eukaryotic elongation factor 2 (eEF‐2) uses transfer of this group in a non‐standard radical reaction (Dong *et al*., 2017a). It may also be worth investigating whether there exist protein modifications (or modifications of metabolites other than polyamines) that use aminopropyl rather than methyl groups. These families of group transfers all produce methylthioadenosine (MTA). Here, we explore the details of the fate of this key MSP compound.

Finally, the third group transfer, the ribosyl transfer that results in epoxyqueuine synthesis on the anticodon of specific tRNAs, does not draw on the methionine pool. It regenerates methionine and gives adenine as a by‐product (Iwata‐Reuyl, 2003). Two further spontaneous AdoMet reactions should also be considered as inputs in a MSP, as they may occur within cells. First, an intramolecular cleavage reaction produces homoserine lactone and MTA. This reaction is exploited in bacteria such as those belonging to the proteobacteria to produce quorum‐sensing (QS) mediators (Dong *et al*., 2017b). Second, a hydrolysis reaction gives adenine and *S*‐pentosylmethionine derivatives, where the pentosyl group represents a variety of ribosyl isomers (Vinci and Clarke, 2007). The fate of homoserine lactones has been well‐studied previously (Dong *et al*., 2001, 2018; Mochizuki, 2001), and we dwell here on that of MTA. In contrast, fine exploration of *S*‐pentosylmethionine compounds metabolism still needs to be developed, in particular with the fate of the sulphur atom in mind. As a possible start point, we note that these compounds are directly related to *S*‐ribosylhomocysteine, which is used as a precursor of autoinducer‐2 (AI‐2) (Fig. 1, red arrows) via the action of LuxS (Han *et al*., 2017). Besides being recycled via AdoHcy, AdoMet cycles back to methionine, forming a full MSP, as we now see.

## General features of the standard aerobic MSP

**Figure 3:**
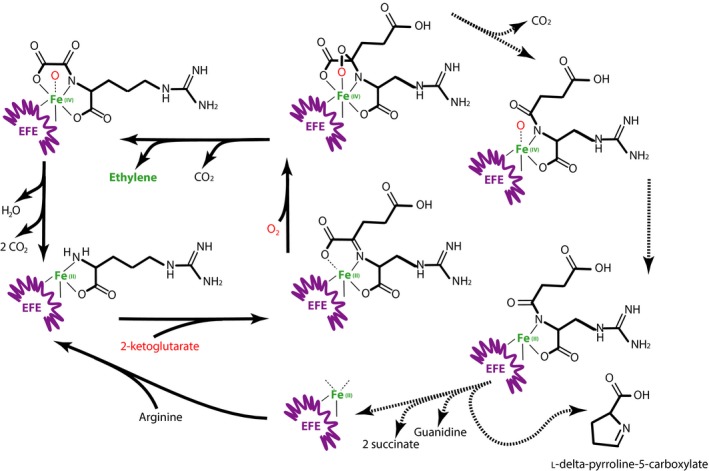
2‐Ketoglutarate ethylene production. Two parallel oxygen‐dependent reactions occur, catalysed by the EFE enzyme. In ethylene synthesis, arginine acts as a cofactor of the reaction. A parasitic reaction (dotted arrows) destroys it with the formation of guanidine and l‐delta‐pyrroline‐5‐carboxylate.

While MTA has been discovered as a by‐product of ethylene production in plants (Fig. 1 dotted blue arrows), the existence of similar pathways in microbes is still open to questions (Billington *et al*., 1979; Fukuda *et al*., 1989; Miller *et al*., 2018a). Sometimes linked to methionine, pathways that produce ethylene often do not produce MTA. For example, a known source of ethylene, present at a low level even in some *E. coli* strains, is the deamination product of methionine, KMBA, generated under nitrogen limitation. Here, ethylene is made together with carbon dioxide and methanethiol. However, the actual biochemical process has not yet been fully deciphered (Mansouri and Bunch, 1989; Shipston and Bunch, 1989). A more productive pathway was identified both in *Pseudomonas syringae* bacteria and in *Penicillium digitatum* fungi. This pathway did not depend on methionine but used an iron‐containing dioxygenase, named EFE, with 2‐ketoglutarate, dioxygen and arginine as substrates. It produced ethylene, succinate, guanidine, carbon dioxide and l‐delta‐1‐pyrroline‐5‐carboxylate. This enzyme is related to 1‐aminocyclopropane‐1‐carboxylate (ACC) oxidases, forming a complex family (Eckert *et al*., 2014). EFE catalysed two different reactions in a 2:1 ratio. In a first reaction, usually repeated twice, arginine remains bound as a cofactor while two 2‐ketoglutarate molecules are converted to six CO_2_ and two ethylene. In the second, parasitic, reaction, both 2‐ketoglutarate and arginine are consumed to yield l‐delta‐1‐pyrroline‐5‐carboxylate, guanidine, succinate and CO_2_ (Fig. 3; Fukuda *et al*., 1992). That this family of reactions is much more than anecdotal is supported by the fact that many bacterial genomes code for specific guanidine exporters, while pathways leading to guanidine production have seldom been identified (Borriss *et al*., 2018). The function of ethylene in these organisms is, however, not properly understood. In general, however, bacteria manipulate ethylene production arising from plant precursors. To be sure, many bacteria degrade ACC via a surface deaminase (Ali and Kim, 2018; Nascimento *et al*., 2018), and while fungi such as *Penicillium citrinum* code for an ACC synthase which may produce MTA, they degrade it into 2‐ketobutyrate and ammonia (Kakuta *et al*., 2001).

The fact is, as we will see below, that ethylene is significantly produced in bacteria under anaerobic conditions via a completely different set of reactions that, nevertheless, proceed via MTA, but through steps common to pathways generally unrelated to the standard MSP that we now consider. Figure 1 depicts this standard MSP arranged at its core as in *B. subtilis* (Sekowska and Danchin, 2002; Ashida *et al*., 2003). It is worth noticing that this arrangement of the MSP is also remarkably similar to that found in human cells (Albers, 2009). It can be split into five parts: synthesis of AdoMet (red arrows, just discussed), production of MTA (blue arrows), conversion of MTA into methylthioribulose‐1‐phosphate (purple arrows), conversion into KMBA (orange arrows) and finally transamination of KMBA to methionine (black arrows).

### Production of MTA (Fig. 1, blue arrows)

As remarked in the previous section, the direct aminocarboxypropyl group transfer represents only a minor source of MTA. Besides tRNA U34 modification or diphthamide synthesis, both modifications of macromolecules – hence removing just a tiny amount of the AdoMet supply – it is also involved in synthesis of metabolites such as betaine glycerolipids that contain an ether‐linked betaine moiety. These unusual lipids are present in lower eukaryotes such as algae, bryophytes, fungi and some primitive protozoa and in photosynthetic bacteria. They are synthesized in bacteria via the action of two enzymes, BtaA, making diacylglycerol‐*O*‐homoserine from 1,2‐*sn*‐diacylglycerol and producing MTA, and BtaB, used three times, producing the final product diacylglycerol‐*O*‐(*N,N,N*)‐trimethyl‐homoserine (Riekhof *et al*., 2005).

By contrast, the major source of MTA comes from aminopropyl transfer after decarboxylation of AdoMet (Fig. 2, green group, see Sekowska *et al*., 2000b). The fact is that synthesis of polyamines, metabolites omnipresent at high concentration, provides the dominant MTA supply in most organisms. Polyamines interact with nucleic acids and interfere with the macromolecule biosynthesis machinery. They scavenge radicals. Yet, their ultimate function remains fairly enigmatic (Cohen, 1998). Specifically, aminopropylation of putrescine into spermidine and of spermidine into spermine are the most prevalent reactions that generate MTA (Knott, 2009). Polyamines also play a key role to support life at high temperature, and thermophilic organisms use large polyamine molecules that derive from amines further modified by aminopropylation (Hidese *et al*., 2017). These latter organisms, as we see repeatedly below, provide excellent models for the biochemistry of the MSP enzymes, which should be considered in priority for biotechnological applications. An example of this theme can be seen in the way polyamines are used in diatoms, which make very long chain polyamines, composed of iterated aminopropyl units as scaffolds for silaffins building up their silica glass skeleton (Michael, 2011).

Synthesis of quorum‐sensing (QS) molecules belonging to the homoserine lactone family (AI‐1) is yet another widespread process that produces MTA routinely as a by‐product. QS signals using AI‐1 molecules are common among many species of proteobacteria (Swift *et al*., 1993; Liu *et al*., 2018). They consist of cell‐permeable fatty acyl‐homoserine lactones. The acyl chain length of these molecules varies between 4 and 18 carbon atoms. Depending on the species, it also differs in backbone saturation or oxidation state at the beta‐carbon. It may also comprise aryl groups. LuxI synthases catalyse the transfer to AdoMet of an acyl group bound to acyl carrier protein (ACP) from fatty acid biosynthesis (Dong *et al*., 2017b). The LuxI family is among the most widespread and the most widely studied AI‐1 synthases. The enzyme from *Vibrio fischeri* producing *N*‐3‐oxohexanoyl‐l‐homoserine lactone was the first enzyme of this family to be identified (Dunlap and Kuo, 1992). MTA is a product of the reaction (Fig. 1, light blue arrows section).

Finally, an enigmatic role of AdoMet, also a source of MTA, appears in the pathway for biotin biosynthesis in a number of bacterial strains. This pathway requires an amino‐group transfer to 8‐amino‐7‐ketononanoate to form 7,8‐diaminopelargonate, a precursor of biotin (Breen *et al*., 2003). Quite surprisingly – knowing the metabolic cost and regulatory role of AdoMet – it is its methionine amino‐group which is used in the process (Fig. 2, pink group), giving *S*‐adenosyl‐4‐methylthio‐2‐ketobutanoate (AdoKMBA) as a by‐product (Stoner and Eisenberg, 1975). In *B. subtilis*, by contrast, lysine is used to this aim (Van Arsdell *et al*., 2005), which makes better sense to the human mind, thus showing that we miss the understanding of an important functional feature of biotin biosynthesis and role. Using AdoMet is the more enigmatic because the reaction produces this dead‐end rare metabolite, AdoKMBA, that needs to be degraded or excreted (Mackie *et al*., 2013). Many of the enzymes that cope with AdoMet or MTA might be involved in degradation of this compound, but the by‐products of the reaction will often be reactive and toxic. A relevant C‐S lyase might give KMBA and 5′deoxyadenosine, or MTA and 2‐ketobutyrate, while the spontaneous fate of the molecule would make 2‐ketobut‐3‐enanoate, that, with its conjugated double bonds, is likely to react with thiols or amino groups. Biotin synthase is an AdoMet radical enzyme (Choi‐Rhee and Cronan, 2005), and the involvement of AdoMet here might suggest that this metabolic pathway is compartmentalized (de Lorenzo *et al*., 2015). Indeed, the fate of deaminated AdoMet interfered with metabolic modelling in a systems biology model of compartmentalized metabolism: Analysis of *Buchnera aphidicola* endosymbiosis asked for addition of an efflux reaction for AdoKMBA which is produced but not consumed in the known biotin biosynthesis pathway (Thomas *et al*., 2009).

With reference to metabolic engineering, we must add a final comment to the understanding of this stage of the MSP. As a consequence of its pervasive presence, MTA regulates, directly or indirectly, expression, activity or both, of enzymes involved in methionine, AdoMet or polyamine biosynthesis (Anashkin *et al*., 2017). Being similar to AdoMet, but smaller, MTA often interferes with AdoMet synthesis or other processes (see for example Pang *et al*., 2017), with a variety of consequences on methionine availability, including inhibition of virulence of pathogens and production of antibiotics (Tojo *et al*., 2014; Wang *et al*., 2017; Bourgeois *et al*., 2018). The outcome of this constraint is that the level of the molecule is generally kept low in cells. Besides possible active excretion, this is the result of the catabolic reactions that we now describe.

### Production of MTRu‐1‐P (Fig. 1, purple arrows)

The immediate fate of MTA differs in different organisms. It has first to be converted into MTR‐1‐P to initiate a MSP. The best energy‐saving way to make MTR‐1‐P is *N*‐ribosyl phosphorolysis of MTA, with adenine as a by‐product. Interestingly, unknown to the majority of investigators (see e.g. Cacciapuoti *et al*., 2003), an enzyme with MTA phosphorolysis activity had already been discovered in 1979, before the discovery of the MSP, in the thermophilic archaeon *Caldariella acidophila* (Cartenì‐Farina *et al*., 1979). This activity looked straightforward, as purine nucleoside phosphorylases make a very large class of enzymes (Bzowska *et al*., 2000) that is split into several families, one of which similar to nucleosidases (Pugmire and Ealick, 2002). In archaea such as *Methanocaldococcus jannaschii* which metabolize MTA via phosphorolysis, MTA phosphorylase MtiP is highly promiscuous (Miller *et al*., 2018b). However, in many organisms, the family has been differentiated into guanine and adenosine phosphorylases, as illustrated for example in *Thermus thermophilus* bacteria (Tomoike *et al*., 2013). The process of activity specification is sometimes even further developed, as in *Mycobacterium smegmatis*, where there are two adenosine phosphorylases, with a form specific for MTA phosphorolysis (Buckoreelall *et al*., 2011). As illustrated with *Mycobacterium tuberculosis*, this may account for the exquisite sensitivity of the organism to methionine deficiency, showing that the MSP is the key to understand methionine homeostasis (Berney *et al*., 2015).

The lesson from these and related works is that there is a considerable range of nucleoside phosphorolysis activities that could be used as a rich mine for developing unique processes of metabolic engineering, or for uncovering novel drug targets. Nucleoside phosphorylases from *E. coli* or *B. subtilis* have previously been used for the synthesis of pharmacologically active compounds, but many more organisms may be used in a variety of conditions to develop microbial synthesis of nucleoside analogues (Almendros *et al*., 2012). Another lesson is that we should not forget that survey of old work may be of considerable value for preparing the future. This enzyme variety, already considerable, has been even explored by living organisms in further directions. Indeed, for reasons that need to be better understood, *Pseudomonas aeruginosa* possesses an unusual pathway for MTA degradation, involving prior to phosphorolysis a deamination step of MTA to 5′‐methylthioinosine (MTI, Guan *et al*., 2011), releasing hypoxanthine, not adenine and MTR‐1‐P as by‐products (Guan *et al*., 2012). Remarkably, MTI is similar to futalosine, the precursor of menaquinone in a family of pathways that have been discovered recently (Dairi, 2012, Fig. 4). The futalosine pathway begins with steps similar to those involving the MTA thiomethyl group but with a 3‐carboxyphenyl‐methylene‐ketone group instead (Kim *et al*., 2014). It appears that, because MTA displays a reactive group smaller than that of futalosine, the MSP may have been recruited from an alternative pathway of menaquinone biosynthesis. In line with this view, a futalosine‐based menaquinone pathway exists also with adenine instead of hypoxanthine as the start point nucleoside (Arakawa *et al*., 2011; Goble *et al*., 2013). At least three different futalosine‐related pathways exist in bacteria, which make it difficult to infer reliable origins for the fate of these nucleosides (Zhi *et al*., 2014). Ancient enzymes, which exchanged readily their genes by horizontal transfer, were likely promiscuous, progressively being shaped into specific activities as the niche of their hosts became narrower.

**Figure 4:**
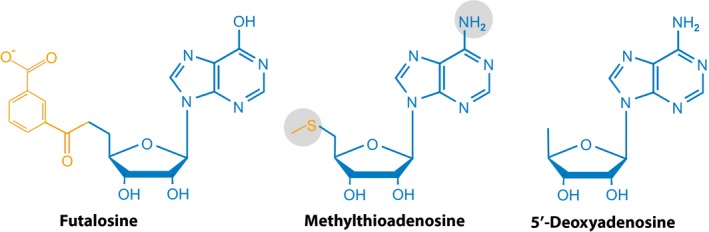
Methylthioadenosine and its related compounds. Futalosine is the inosine analogue used in the reference futalosine‐dependent menaquinone pathway. Its amino‐ form is sometimes used.

Besides a direct course involving phosphorolysis by MtnP, the path from MTA to MTR‐1‐P may also be indirect. To be sure, in the first cells where the MSP was explored, MTR‐1‐P resulted from the consecutive action of two enzymes that were subsequently characterized biochemically, a nucleosidase, MtnN, producing MTR (Mishra and Ronning, 2012), and a kinase, MtnK (Ku *et al*., 2007). In bacteria, the nucleosidase recruited in this pathway was the same promiscuous enzyme that hydrolysed *S*‐adenosylhomocysteine in the process of AI‐2 generation (Fig. 1, red arrows). As a consequence, the activity of MtnN interfered with AI‐2 synthesis and biofilm formation in a variety of bacterial species (Kang *et al*., 2014b; Thomas *et al*., 2015; Han *et al*., 2017). This intermediate step allowed leakage of MTR out of the cell if needed (for MTR transporters identification, see experiments reported in the supplementary text to Borriss *et al*., 2018). In some organisms, such as *E. coli*, MtnK is missing and MTR is excreted as a dead‐end metabolite (waste, signalling molecule or sulphur level buffering compound). This latter activity may explain why, while it already contained a cytoplasmic MTA nucleosidase, *Aeromonas hydrophila* has a counterpart located in the periplasm. This also suggests that MTA can be available, possibly as a signalling molecule, in certain environments (Xu *et al*., 2017).

At this point, we have reached the crux of the MSP. Unequivocally, besides MTA, the pivotal metabolite of the MSP is MTRu‐1‐P, the immediate downstream metabolite of MTR‐1‐P. This metabolite results from the action of MTR‐1‐P isomerase MtnA (Tamura *et al*., 2008), an enzyme with a widely conserved structure (Saito *et al*., 2007) but promiscuous, as shown in *M. jannaschii* where it acts both on the MSP and on its 5′‐deoxyadenosine catabolism paralogous pathway (Miller *et al*., 2018b). Perhaps because of the importance of this step, the human enzyme MRI1, initially identified by a cancer‐related phenotype, is highly similar to this widespread enzyme (Kabuyama *et al*., 2009). Finally, in line with the consistency of this part of the pathway relating MTA to MTRu‐1‐P, MtnK and MtnA are fused together as a bifunctional protein MtnAK in *Tetrahymena thermophila* (Nakano *et al*., 2013b).

### Production of KMBA, the keto precursor of methionine (Fig. 1, orange arrows)

The structure MTRu‐1‐P is related to that of many metabolites of the core carbon metabolism. As a consequence, many different enzymes may have been recruited via initial moonlighting to orient its chemical fate, depending on the organism's niche. We deal in this paragraph with the three parallel known routes that end up in the standard MSP by recovering methionine via KMBA. Other non‐canonical routes will be described subsequently.

All begin with a dehydratase, MtnB, which can act in isolation or associated to a series of domains that channel the products of the dehydration reaction to its ultimate step, a dioxygenase. This dehydratase is widespread in prokaryotes. It belongs to the aldolase class II family which may rapidly evolve via its moonlighting activities (Lee *et al*., 2017). MtnB produces 2,3‐diketo‐5‐methylthio‐1‐phosphopentane (Ashida *et al*., 2008). It has also close counterparts in eukaryotes (Pirkov *et al*., 2008). Gene APIP, for example, is the counterpart in *H. sapiens* where it is involved in the control of the key process of apoptosis (Kang *et al*., 2014a). Two different types of reactions have been recruited to generate 1,2‐dihydroxy‐3‐keto‐5‐methylthiopentene. The reactions first described in molecular details came from the study of *B. subtilis*. They are not the most widespread ones, however. In this bacterium, as we saw in the introduction, a RuBisCO‐like enzyme, MtnW, synthesizes 2‐hydroxy‐3‐keto‐5‐methylthiopentenyl‐1‐P, which is subsequently dephosphorylated by phosphatase MtnX (Ashida *et al*., 2003). By contrast, in most organisms, the widespread enolase/phosphatase MtnC is a bifunctional enzyme. It yields 1,2‐dihydroxy‐3‐keto‐5‐methylthiopentene directly, with phosphate as a by‐product after channelling the substrate's intermediary products within the enzyme (Sekowska *et al*., 2004; Zhang *et al*., 2004). A counterpart is present in eukaryotes, and the plant enzyme has been studied in some details (Wang *et al*., 2005; Pirkov *et al*., 2008; Pommerrenig *et al*., 2011).

At this point, the dioxygen‐dependent step of the standard MSP comes into play, with dioxygenase MtnD generating, besides KMBA, the one‐carbon formate as a by‐product. This dioxygenase is ubiquitous in organisms living in the presence of oxygen. It has the remarkable property that its specificity depends on the metal present at its catalytic centre (Deshpande *et al*., 2017). When iron is not limiting, KMBA and formate are produced. By contrast, when nickel replaces iron, the products are 3‐methylthiopropionate, formate and carbon monoxide (Sparta *et al*., 2013). Methylthiopropionate has long been identified as a product of methionine catabolism (after decarboxylation of KMBA), a route complementary to that allowed by methioninase when methionine is present in excess in cells (El‐Sayed, 2010). Its fate has seldom been studied, except in human cells (Steele and Benevenga, 1979), where an unknown C‐S lyase generated methanethiol which was further metabolized either via transsulphuration or via oxidation (Fig. 1, black dashed line arrows, Blom *et al*., 1988). In some unicellular organisms, such as *T. thermophila*, dehydratase MtnB and dioxygenase MtnD are fused together into a MtnBD fusion protein (Nakano *et al*., 2013b). Quite remarkably, this protein has further multifunctional activity as it is able to do in one step what yeast does in three, since it can rescue yeast gene knockouts of *MDE1*(*mtnB*), *UTR4*(*mtnC*) or *ARD1*(*mtnD*) (Salim *et al*., 2009). At this point, the MSP has produced KMBA, the ketoacid precursor of methionine by transamination. We now see that the pathway evolved with a twist: while transamination is standard for amino acids, the route taken by methionine is unique, possibly because keeping sulphur in a reduced state is costly.

### Making the MSP cycle irreversible (Fig. 1, black arrows)

In general, the presence of dioxygen goes against reduced sulphur availability because oxygen tends to react with sulphur while the energy cost of reducing sulphonates is high. A remarkable way out is well illustrated in *B. subtilis*, which as an epiphyte is doomed to meet frequently large concentrations of dioxygen. In this organism, several oxygen‐dependent cysteine salvage pathways exist that preserve the cysteine thiol group. They should be of great interest as seeds for novel approaches to metabolic engineering of sulphur‐containing compounds, escaping formation of sulphonates despite the involvement of oxygenases (Chan *et al*., 2014; Niehaus *et al*., 2018). Besides cysteine, methionine also contains a reduced sulphur atom and must be salvaged. Being the first residue of all proteins, it is essential that it remains readily available where translation operates. This requires turnover of this first residue of polypeptides, a process that affects about half of newly synthesized ones (Wingfield, 2017) yet is insufficient to recycle enough methionine for its overall involvement in metabolism. As a consequence, because the methionine supply is limiting under conditions where biochemical reactions are generally reversible, this constraint requires that methionine salvage processes are organized in such a way as to prevent methionine degradation. This ratchet‐like process, which extends far beyond the bacteria domain – being of key importance in *Homo sapiens* for example (Mary *et al*., 2012) – is a prominent feature of the canonical MSP.

**Figure 5:**
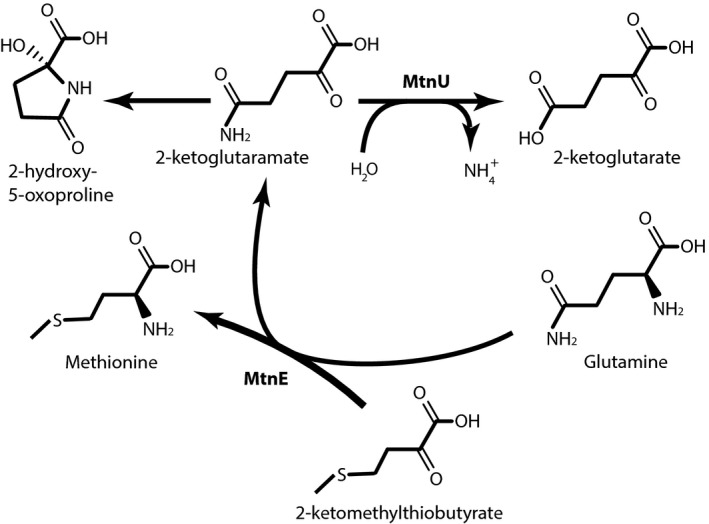
The ketoglutaramate ratchet. Transamination of ketoacids with glutamine as an alpha‐amino‐donor results in the formation of 2‐ketoglutaramate, which must be deaminated by an omega‐amidase.

As a prevailing rule, the amino group donor of anabolic transamination reactions is the alpha‐amino group of aspartate or glutamate. This makes sense in that the product of the reaction, oxaloacetate or 2‐ketoglutarate, respectively, directly feeds into the citrate cycle. In complement, the amido group of asparagine or glutamine are used in a variety of reactions requiring transfer of an amido group to substrates that are not amino acids, while generating, again, oxaloacetate or 2‐ketoglutarate (Raushel *et al*., 1999). By contrast, usage of the alpha‐amino group of asparagine or glutamine in a transamination reaction is surprising as the reaction generates 2‐ketosuccinamate or 2‐ketoglutaramate, respectively (Meister, 1953). These compounds, which are omitted from standard metabolic charts, are quite reactive. They may cyclize spontaneously, possibly leading to metabolic accidents (Danchin, 2017). For example, 2‐ketosuccinamate tends to dimerize, with subsequent ring closure, dehydration, aromatization, deamidation and decarboxylation, leading to a plethora of heterocyclic compounds (Cooper *et al*., 1987). In the same way, ketoglutaramate may cyclize spontaneously into the lactam compound 5‐hydroxypyroglutamate (2‐hydroxy‐5‐oxoproline), the fate of which has seldom been explored (Fig. 5, Cooper and Kuhara, 2014). Importantly, maintaining the intracellular content of ketoglutaramate at a low level via hydrolysis of the amido group will render the glutamine transaminase reaction irreversible (Hersch, 1971). This can be performed by omega‐amidases belonging to the large nitrilase superfamily (Huebner *et al*., 2011). For example, an omega‐amidase was found to be involved in the degradation of glutamine in *Neurospora crassa* (Calderon *et al*., 1985). Curiously, however, the role of these enzymes has only been linked to the MSP recently (Krasnikov *et al*., 2009b). Whereas removal of the ketoglutaramate amido group has been documented as key for an efficient MSP in the case of *B. subtilis* (Belda *et al*., 2013), other roles such as that in the catabolism of nicotine had usually been emphasized, while the authors noticed, however, that the corresponding genes were often located in MSP operons (Cobzaru *et al*., 2011). We see therefore that using glutamine as an alpha‐amino‐donor is a way to render amino group transfer irreversible. This may go far beyond the MSP.

Taken together these observations – that correlate with involvement of important processes such as tumour suppression in animals (Krasnikov *et al*., 2009a) – complete the MSP by forcing it in the direction of methionine synthesis in a ratchet‐like manner, via hydrolysis of ketoglutaramate (Danchin and Sekowska, 2014). This may account for the ubiquitous presence of omega‐amidases, the role of which is still largely ignored in metabolic engineering.

## Alternate fates of MTRu‐1‐P and its by‐products

As we just discussed, the steps of the MSP leading to production of MTRu‐1‐P from MTA match a section of recently identified pathways of menaquinone biosynthesis: synthesis of carboxynaphtoquinol via futalosine (Zhi *et al*., 2014). Remarkably, a second section of menaquinone synthesis, its isoprenoid skeleton synthesis, is also involved in the scavenging of MTRu‐1‐P in a variety of organisms. Indeed, besides its involvement in the standard aerobic MSP just described, the crossroad metabolite MTRu‐1‐P is also at the origin of other pathways that impact the global sulphur cycle, without systematically leading to methionine salvage, however. In terms of evolution, it seems quite remarkable that, again, the MSP enters menaquinone biosynthesis (Erb *et al*., 2012). This opens up interesting questions about prebiotic links between sulphur metabolism, complex lipids metabolism and electron transfers. These pathways produce isoprenoids or ethylene (under anaerobic conditions) along routes that we now describe.

### A pathway input to isoprenoid biosynthesis (Fig. 1, green arrows)

Hints that pathways differing from the standard MSP could exist were revealed by the study of *R. rubrum* and related organisms. The first of those alternative pathways was found to be oxygen‐independent, working both in aerobic and anaerobic conditions. As a start, polyamine biosynthesis in *R. rubrum* produced MTA and MTRu‐1‐P from AdoMet as in other organisms. Also, as reported in the introduction, early complementation studies suggested that methionine could be an ultimate product of a pathway possibly similar or identical to previously known MSPs. Further work, however, showed that this species lacked the classical MSP. Instead, a MTRu‐1‐P 1,3‐isomerase, highly similar to RuBisCO/MtnW, catalysed isomerization of 5‐methylthio‐d‐ribulose‐1‐phosphate (MTRu1‐P) to a 3:1 mixture of 1‐methylthio‐d‐xylulose‐5‐phosphate (MTXu‐5‐P) and methylthio‐d‐ribulose‐1‐phosphate (note the ‘inverted’ numbering, discussed in Imker *et al*., 2008). In turn, this mixture entered a reductive pathway, the details of which are still being explored (Erb *et al*., 2012; North *et al*., 2017), involving a MTXu‐5‐P methylsulphurylase, a member of the cupin superfamily (Uberto and Moomaw, 2013). This enzyme produced methanethiol and 1‐deoxy‐d‐xylulose‐5‐P (DXP, Warlick *et al*., 2012b), the key precursor of isoprenoids in bacteria (Dickschat, 2011). Subsequently, a similar pathway was identified in *Rhodopseudomonas palustris* (Miller *et al*., 2018a).

Methanethiol production in this pathway is still under investigation. It required the presence of an active thiol. Interestingly, this essential substrate of the reaction is doomed to differ in different microbial organisms [glutathione frequently (Anderson, 1998; Pocsi *et al*., 2004; Smirnova and Oktyabrsky, 2005), but bacillithiol in Firmicutes (Chandrangsu *et al*., 2018), and mycothiol in Actinobacteria (Fahey, 2013)]. In terms of biochemical properties (and gene annotations, too), this implies that the enzymes involved are not true orthologues but generally paralogues, with specific features meant to accomodate the various thiol substrates, making their accurate identification difficult. Generation of an authentic salvage pathway (i.e. ending in methionine in a stoichiometric manner) further depends considerably on the availability of the reduced forms of these thiols, which will follow closely the redox and free radical environments of the organism. As a consequence, it seems likely that methanethiol will be processed by means that, often, will not automatically lead to methionine salvage.

Indeed, the known fate of methanethiol is highly variable, depending on the organism and on its particular niche (Weise *et al*., 2012). This highly volatile molecule may evade the cell as a gas, be oxidized to methane sulphonate (Vermeij and Kertesz, 1999), or, indeed, be recovered for methionine salvage via *O*‐acyl‐homoserine (*O*‐acetyl‐ or *O*‐succinyl‐ as preferred protecting groups) by cystathionine gamma‐synthase or related enzymes (Kiene *et al*., 1999, with considerable expected variations in acylation, depending on the species, see Bastard *et al*., 2017). As an example, assimilation of methanethiol and dimethyldisulphide has been studied in some species, *Corynebacterium glutamicum* in particular (Bolten *et al*., 2010). Methanethiol is incorporated as an entire molecule, adding the terminal S‐CH_3_ group to *O*‐acetylhomoserine. In this reaction, methionine is directly formed. MetY (*O*‐acetylhomoserine sulphhydrylase) was identified as the enzyme catalysing the reaction. The extent of this pathway in methionine salvage – likely to have been of prime importance in the early times of life, before the dioxygen rise – is, however, expected to be moderate, as methanethiol is often exported as waste rather than recovered for anabolism. This is shown for example in the metabolism of *Pelagibacter ubique* (Sun *et al*., 2016).

Nevertheless, it is tempting to note that methionine salvage by routes related to this isoprenoid pathway may be a key feature of marine environments where sulphur is limiting (Mazel and Marliere, 1989). To be sure, dimethyl sulphide is a key regulator of marine life. It is metabolized via a variety of pathways that may be relevant to the pathways discussed here. In particular, its precursor, dimethylsulphoniopropionate (DMSP), is degraded into methanethiol and also to DMS via promiscuous enzymes of the cupin family that are likely to be relevant in the present context (Lei *et al*., 2018). DMSP is produced in abundant quantities by marine surface‐water phytoplankton (Tan *et al*., 2013). The catabolism of this compound is an important source of carbon and reduced sulphur for marine bacteria and other organisms. Indeed, sulphur availability is often limiting in marine environments, and methanethiol assimilation is an important step of DMSP assimilation, illustrated for example in *Synechococcus* sp. (Malmstrom *et al*., 2005). The enzyme (methylthio)acryloyl‐CoA hydratase, DmdD, a member of the crotonase superfamily, catalyses the last step in the methanethiol pathway of DMSP catabolism: 3‐(methylthio)acryloyl‐CoA + 2 H_2_O = acetaldehyde + methanethiol + CoA + CO_2_ (Tan *et al*., 2013). This invites us to try and document further this process, as it is likely to have a key role in the general sulphur cycle on Earth. This is also a feature that should be taken into account for choosing novel bacterial chassis in particular alpha‐proteobacteria and possibly cyanobacteria for metabolic engineering of alkyl‐sulphur‐containing compounds. Methanethiol can also be produced by further pathways that we now consider.

### A pathway for anaerobic ethylene synthesis in bacteria

*Escherichia coli* generally does not have a functional MSP, exporting MTR as waste (Hughes, 2006). It came out as a surprise, therefore, that strain S88 (O45:K1:H7), an extra‐intestinal pathogenic *E. coli* strain (ExPEC) isolated from the cerebrospinal fluid of a late onset neonatal meningitis case in France in 1999 appeared, besides the omnipresent nucleosidase MtnN, to code for counterparts of MtnK, MtnA and MtnB (encoded in an operon ending with a transporter gene), but no downstream enzymes of the MSP (Touchon *et al*., 2009). Halting the MSP at a point where MTRu‐1‐P would be produced looked metabolically impossible because of the high reactivity of the compound. This suggested therefore that this intermediate was metabolized via an unknown pathway or that it was involved in a paralogous pathway, as illustrated previously with the example of *M. jannaschii*.

A solution to this challenging question may come from the identification of a second catabolic pathway involving MTRu‐1‐P, found both in *R. palustris* and *R. rubrum* (North *et al*., 2017). This pathway, named the ‘DHAP‐ethylene shunt’ is exclusively anaerobic (Miller *et al*., 2018a). Remarkably, besides methanethiol, it produces ethylene in a way totally different from that found in plants, starting from MTRu‐1‐P (Fig. 1, dark blue broken line arrows, followed by light green lines for the strictly anaerobic section). There, MTRu‐1‐P is first cleaved into dihydroxyacetone phosphate (DHAP) and 2‐(methylthio)acetaldehyde (North *et al*., 2017), using a class II aldolase‐like protein that belongs to a family of promiscuous DHAP‐dependent enzymes widely spread in bacteria (Laurent *et al*., 2018). A similar pathway may exist in other bacteria as well, but this has not been explored yet. For example, *E. coli* S88 possesses several sugar(bis)phosphate aldolase‐like enzymes with no counterparts in *E. coli* K12 (e.g. ECS88_3638, ECS88_4074; Touchon *et al*., 2009) which would readily fit in the picture (Lee *et al*., 2017). Also, aldolases unexpectedly involving sulphur‐containing sugars have been described, such as the 6‐deoxy‐6‐sulphofructose‐1‐phosphate aldolase (SquT, b3881 in *E. coli* K12) of the widely spread sulphoquinovose degradation pathway (Denger *et al*., 2014). However, the strongest argument pleading in favour of a paralogous pathway in strain S88 is the following. The gene product annotated as the counterpart of MtnB (ECS88_4903) is in fact remarkably similar to aldolase Ald2 that has been proposed to enter the anaerobic ethylene pathway in *R. rubrum* (North *et al*., 2017, Fig. 6).

**Figure 6:**
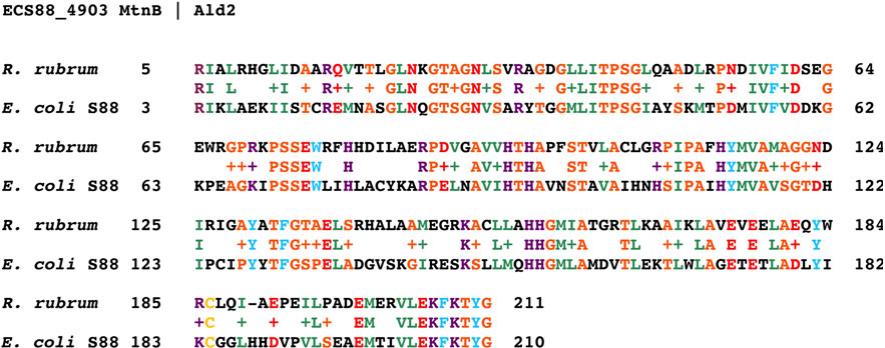
Alignment of *Escherichia coli* S88 and *Rhodospirillum rubrum* aldolases. Conserved residues (+) follow Margaret Dayhoff's related amino acids rule: ASTPG (orange), MLIV (green), HKR (dark purple), DENQ (red), FWY (blue), C (yellow).

It is worth emphasizing that promiscuity of these enzymes is such that simple mutations can re‐route them to novel pathways, including to metabolism of 5′‐deoxyadenosine (5′dAdo), as we see below, or to an isoprenoid synthesis pathway (King *et al*., 2017). This versatility may explain why the actual way ethylene is produced in *R. rubrum* is still a matter of speculation. Only explicit biochemical identification of the activities involved will allow investigators to concretize the key elements of the pathway. In this organism, the 2‐(methylthio)acetaldehyde formed by the aldolase is subsequently reduced by an alcohol dehydrogenase – there are many candidates with this activity in most genomes – to 2‐(methylthio)ethanol. Here, this metabolite is further metabolized by unknown enzymes to recycle the methylthio‐ moiety, while generating ethylene (North *et al*., 2017; Miller *et al*., 2018a). The methanethiol product made here – by a still unknown anaerobic pathway – could be directly utilized by *O*‐acyl‐l‐homoserine sulphhydrylase to regenerate methionine as described previously. Phylogenetic analyses suggest that the pathways are widespread among anaerobic and facultatively anaerobic bacteria from soil and freshwater environments.

It may be relevant to note here that, by contrast, a variety of routes exist to metabolize methylthioethanol. It can be methylated, for example (Carrithers and Hoffman, 1994). Also, many bacteria produce (methylthio)acetaldehyde as a methionine degradation product via KMBA, not for methionine salvage. For example, in *Lactococcus lactis*, methionine is degraded after deamination producing (methylthio)acetaldehyde, which can be reduced to methanethiol. However, the reaction requires aerobic conditions as it results from oxidative cleavage of the tautomeric form of KMBA, producing the aldehyde and oxalate or carbon dioxide (Bonnarme *et al*., 2004). The situation in *R. palustris* further illustrates how variation on this catabolic theme, in aerobic conditions, could result in methionine salvage without ethylene production. Indeed, besides the strictly anaerobic DHAP‐ethylene shunt, *R. palustris* displays a DHAP‐methanethiol shunt in the presence of oxygen (Miller *et al*., 2018a). The first steps of this partially redundant pathway are identical to those of the DHAP‐ethylene shunt, but, rather than generate ethylene, they produce directly methanethiol, which possibly goes back to methionine in a MetY‐dependent process similar to that described previously. Work needs to be developed to unravel the details of these partially redundant pathways, as well as understand their functional logic.

A puzzling question remains: An authentic RuBisCO appears to be necessary for anaerobic MTA metabolism in *R. rubrum*, and two homologues exist in *R. palustris*. Complementation studies performed with mutant RuBisCO indicated that RuBisCO is required for MTA metabolism but there is no indication of the actual reaction involved (Miller *et al*., 2018a). It was also demonstrated by complementation that all known forms of authentic RuBisCO could perform this unknown function. Metabolomics studies indicated that this function could be linked to the formation of *S*‐methyl‐3‐mercaptopyruvate and *S*‐methyl‐l‐cysteine (Dey *et al*., 2015). These observations suggest deep links between carbon and sulphur metabolism. It is tempting to relate this observation to the discovery of a role of a class of RuBisCO protein in the ‘reductive hexulose‐phosphate pathway’ discovered in methanogenic archaea (Kono *et al*., 2017). This pathway resembles the photosynthetic Calvin–Benson cycle. However, some reaction steps of the Calvin cycle are replaced by different enzyme‐catalysed steps, indicating that the pathway may be an ancient CO_2_ fixing pathway using RuBisCO, before establishment of photosynthesis. A related observation substantiates this view. The RuBisCO‐like enzymes (RLPs) involved in the catabolic pathway for d‐apiose, – a branched pentose present in rhamnogalacturonan‐II‐containing cell walls of higher plants and in apigalacturonan containing the cell walls of aquatic monocots – have activities reminiscent to those postulated here. As cases in point, the RLPs from *Agrobacterium radiobacter* K84 and *Xanthobacter autotrophicus* ATCC BAA‐1158 catalyse decarboxylation and transcarboxylation/hydrolation of 3‐oxo‐isoapionate‐4‐phosphate (Carter *et al*., 2018). These newly identified pathways may thus be related to the fate of AdoMet after its involvement in the many pathways where it is an essential substrate before dioxygen was widespread.

We have explored how the MTA thiomethyl group is involved in methionine salvage. The adenosyl moiety is yet another costly by‐product that needs to be recycled efficiently, and it is not unlikely that the corresponding pathways are related to one another. We now summarize our knowledge in the domain.

## 5′‐deoxyadenosine metabolism as a paralogous pathway of the MSP

While the fate of MTA has been seen as a biochemical challenge for decades, the fate of another by‐product of AdoMet involvement into many key metabolic pathways seems to have been somewhat overlooked. A large collection of enzymes utilize the high reactivity of radicals for catalysing chemically challenging reactions. Among those, we retain here the radical AdoMet superfamily of enzymes (Shibata and Toraya, 2015). These radical AdoMet enzymes display a considerable variety of radical reactions, based on the way reactive groups are bound to the key sulphur atom (Davis and Boal, 2017). This is reminiscent of the group transfers we have previously discussed (see the biochemical rationale for the standard MSP). To be sure, we have noticed that diphthamide biosynthesis generated MTA via a radical reaction that was at odds with those mediated by the other well‐known radical AdoMet enzymes (Dong *et al*., 2017a). Further, in a few reactions, AdoMet is recycled intact, as is a coenzyme. Yet AdoMet is generally used as a substrate, being irreversibly cleaved with release of methionine at each turnover. Here, we further restrict our analysis to those enzymes that use the AdoCH_2_ radical for their activity (Wang and Frey, 2007), and we retain the enzymes that generate 5′‐deoxyadenosine (5′‐dAdo) at the end of the reaction they catalysed.

5′‐deoxyadenosine is quite similar to MTA and smaller than that molecule (Fig. 4). It can therefore be expected that the initial part of its fate will parallel that of MTA, possibly using a moonlighting activity of the same enzymes. As seen previously, two major pathways will result in production of 5‐deoxyribose‐1‐phosphate and adenine, either combining a nucleosidase and a kinase or using directly a *N*‐riboside phosphorylase. A nucleosidase pathway is explicit in *M. tuberculosis* with MtnN (Rv0091) catalysing 5′‐dAdo hydrolysis. There, 5′‐dAdo is the preferred substrate (Namanja‐Magliano *et al*., 2017) but it could first be deaminated into 5′‐deoxyinosine, as in the futalosine pathway. Downstream, the fate of the 5‐deoxyribose product is, however, generally unknown. Yet, nucleosidases are common in detoxification processes. They produce molecules related to 5‐deoxyribose. For example, 5‐deoxy‐d‐ribitol was found as a major metabolite of 5′‐deoxy‐5‐fluorouridine degradation in rats (Ichihara *et al*., 1985). This implies formation of 5‐deoxy‐5‐fluoro‐ribose, prior to action of a dehydrogenase. Another idea of what could happen can be seen in the catabolism of 5‐chloro‐5‐deoxy‐d‐ribose in the marine actinomycete *Salinispora tropica* by a chlorodeoxyribose‐1‐dehydrogenase producing chloroethylmalonyl‐CoA (Kale *et al*., 2010). Ethylmalonyl‐CoA would then enter an acetate pathway as in *Methylobacterium extorquens* (Schneider *et al*., 2012). Another hint at a role of the nucleosidase in 5′‐dAdo catabolism is provided by a link in *E. coli* between MSP and biotin synthesis. We noted previously that biotin synthase is a radical AdoMet enzyme that inserts sulphur into dethiobiotin to make biotin. The reaction produces 5′‐dAdo. Strains lacking MtnN fail to make biotin and possibly lipoic acid, made by another AdoMet radical enzyme producing 5′‐dAdo as well (Choi‐Rhee and Cronan, 2005).

This is a strong argument for a pathway paralogous to the first steps of the MSP. A promiscuous kinase, related to MtnK could yield 5‐deoxyribose‐1‐phosphate entering a pathway similar to the MSP. This same product is the direct product of a N‐riboside phosphorylase, illustrated by the highly promiscuous primitive enzyme MtiP which has been described in *M. jannaschii*, as we previously discussed. Remarkably the promiscuity of this enzyme is such that it would also accomodate the first steps of the futalosine (or aminofutalosine) pathway, as well as those of the MSP (Miller *et al*., 2018b). This would lead to overlapping paralogous pathways (Chan *et al*., 2014). We can propose that, more often than not, the first steps of 5′‐dAdo degradation will proceed by a futalosine/MSP moonlighting pathway. This is substantiated by the presence of a 5′‐dAdo deaminase in this same organism (Miller *et al*., 2014). MtiP is active with six different purine nucleosides, including forms containing hypoxanthine and therefore able to participate in the MSP, in menaquinone synthesis and in 5′‐dAdo phosphorolysis, possibly after deamination into 5′‐deoxyinosine, as well as in general purine salvage. MtiP generates both 5‐methylthioribose‐1‐phosphate and 5‐deoxyribose‐1‐phosphate as products, from phosphorolysis of 5′‐methylthioinosine and 5′‐deoxyinosine, respectively (Miller *et al*., 2018b). Detailed molecular knowledge of these activities may be of considerable interest for biotechnology approaches. Indeed, making promiscuity explicit is the key to develop novel engineered pathways. Ignoring promiscuity may jeopardize metabolic engineering as a consequence of interference from pre‐existing production of substrates generated within the standard chassis (Saito *et al*., 2007). As a case in point, it was shown that promiscuity of the whole MSP was sufficient to allow ribose conversion to homoserine (Nakano *et al*., 2013a).

At this point, the MSP, futalosine and 5′‐dAdo pathways are expected to diverge, as 5‐deoxyribose‐1‐phosphate appears to be fairly stable (Plagemann and Wohlhueter, 1983). Interestingly, Miller *et al*. proposed that isomerase MJ0454 produces 5‐deoxyribulose‐1‐phosphate in a reaction parallel to that present in the standard MSP (Miller *et al*., 2018b). As a subsequent step, we can propose that an aldolase would produce acetaldehyde and DHAP, in a pathway parallel the anaerobic ethylene pathway (see also the d‐apiose pathway that we just discussed). This hypothesis is strongly supported by the metabolism of fluorometabolites in *Streptomyces cattleya* where a fluorinated derivative of AdoMet is submitted to phosphorolysis. The fluorinated phosphodeoxyribose is then submitted to the action of a 5‐fluoro‐5‐deoxyribose‐1‐phosphate isomerase (Onega *et al*., 2007) and a 4‐fluorothreonine acetaldehyde transaldolase allows the bacterium to generate fluoroacetate in a mimic of the standard metabolism of acetaldehyde (Zhao *et al*., 2012). This pathway was indeed experimentally validated in *Bacillus thuringiensis* after submission for publication of the present review (Beaudoin *et al*., 2018). Another pathway would, in the presence of dioxygen, follow the route of the standard MSP, but the MtnD dioxygenase would now yield 2‐ketobutyrate instead of KMBA. These pathways would allow catabolism of 5‐deoxyribose‐1‐phosphate to enter the standard carbon/amino acid metabolism.

## Concluding remarks: oxygen and metabolic evolution

Methionine and AdoMet are the key metabolites that are involved both in the macromolecule biosynthesis machinery and in intermediary metabolism. Containing sulphur, there are likely to have been present since the origins of the first cells. As a consequence, the metabolic pathways that involved these compounds were first developed in the absence of dioxygen, in a neutral or slightly reducing environment. It is therefore worth emphasizing that methionine recycling and synthesis of mevalonate could be related both in synthesis of the aromatic part of the molecule, via the futalosine pathway, and in the synthesis of isoprenoids. The development of reactions involving the AdoCH_2_ radical is also likely to be ancient, as these reactions allowed the development of difficult chemistry in an anoxic context (Marsh *et al*., 2010). While these reactions often produced 5′‐dAdo, it seems natural that processes allowing scavenging of adenine while recovering the carbon skeleton of the molecule were developed very early on. A general anoxic pathway to this aim has been described in the last section of this article, and we may take it as a start point to understand how it further evolved. Remarkably, enzyme promiscuity would have been easily adapted to recycling of MTA which is an inevitable product of polyamine synthesis, also likely to be ancient metabolites. These overlapping activities appear to have been conserved in *M. jannaschii*, as a set of paralogous pathways driven by a specific set of promiscuous enzymes (Miller *et al*., 2018b). The key enzymes in this family of pathways are purine nucleoside phosphorylase and an aldolase, member of the large ancestral class II superfamily. The downstream strictly anaerobic pathway would have resulted in production of ethylene and methanethiol, which would be used to make methionine from *O*‐activated homoserine. An enigmatic feature of this pathway, reminiscent of primaeval life conditions, remains unsolved at this time: an authentic RuBisCO, presumably involved in carbon dioxide recovery, appears to be essential in the process (Dey *et al*., 2015).

**Figure 7:**
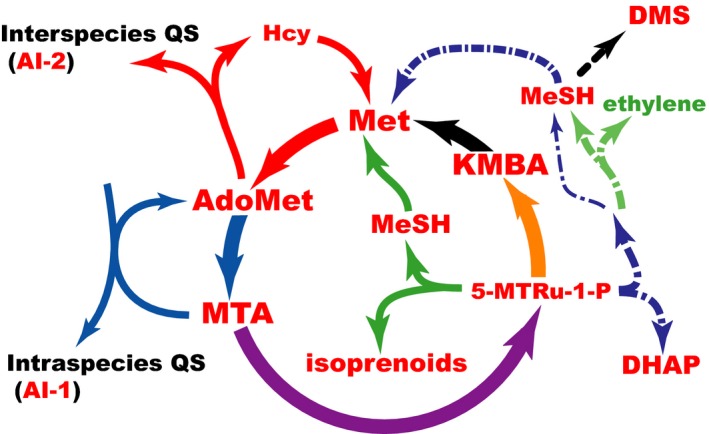
Overview of the MSP. The MSP connects together important functions that drive cellular collective behaviours, such as quorum‐sensing, while regulating sulphur homeostasis in marine (and soil) environments via generation of gaseous molecules.

This pathway, while beneficial in the way it allows methyl‐sulphur recovery, is not very cost effective, metabolically speaking. It suffers significant losses due to the volatile nature of two of its products, ethylene and methanethiol. Invasion of the atmosphere by dioxygen resulted in a variety of chemical possibilities that could not be developed in the absence of this strong ubiquitous electron acceptor, accounting for the role of the MSP in such diverse functions as quorum‐sensing, isoprenoids synthesis and marine or soil production of methanethiol, dimethylsulphide or ethylene (Fig. 7). This has been witnessed in the alteration of metabolic pathways, both in primary and in secondary metabolism that followed this first life‐driven pollution of the Earth (for a discussion see Takemoto and Yoshitake, 2013). We can therefore understand the standard MSP as an improvement of a previous awkward salvage pathway that diverted the function of existing enzymes (see how MtnB is related to the anoxic aldolase of the original pathway) from a role in the absence of dioxygen to its presence. A ratchet mechanism had also to be included in the process so that degradation of methionine, which would be sensitive to the presence of oxygen, would be prevented. The consequence of this setup is of considerable importance for choosing relevant synthetic biology chassis. Indeed, the investigators involved in metabolic engineering must choose, beforehand, their chassis as a function of the planned growth conditions. Interestingly, this may be what evolution found when most cancer cells, which have to multiply fast, hence under low oxygen pressure, deleted genes of their parent's MSP.

## Conflict of interest

None declared.
